# Dual terahertz comb spectroscopy with a single free-running fibre laser

**DOI:** 10.1038/s41598-018-29403-9

**Published:** 2018-07-24

**Authors:** Guoqing Hu, Tatsuya Mizuguchi, Ryo Oe, Kazuki Nitta, Xin Zhao, Takeo Minamikawa, Ting Li, Zheng Zheng, Takeshi Yasui

**Affiliations:** 1 Graduate School of Advanced Technology and Science, Tokushima University, 2-1, Minami-Josanjima, Tokushima, Tokushima, 770-8506 Japan; 2 School of Electronic and Information Engineering, Beihang University, 37 Xueyuan Rd., Beijing, 100191 China; 3 JST, ERATO, MINOSHIMA Intelligent Optical Synthesizer Project, 2-1, Minami-Josanjima, Tokushima, Tokushima, 770-8506 Japan; 4 Graduate School of Technology, Industrial and Social Sciences, Tokushima University, 2-1, Minami-Josanjima, Tokushima, Tokushima, 770-8506 Japan; 5 Collaborative Innovation Centre of Geospatial Technology, 129 Luoyu Road, Wuhan, 430079 China

**Keywords:** Frequency combs, Terahertz optics

## Abstract

Dual terahertz (THz) comb spectroscopy enables high spectral resolution, high spectral accuracy, and broad spectral coverage; however, the requirement for dual stabilized femtosecond lasers hampers its versatility. We here report the first demonstration of dual THz comb spectroscopy using a single free-running fibre laser. By tuning the cavity-loss-dependent gain profile with an intracavity Lyot filter together with precise management of the cavity length and dispersion, dual-wavelength comb light beams with slightly detuned repetition frequencies are generated in a single laser cavity. Due to sharing of the same cavity, such comb light beams suffer from common-mode fluctuation of the repetition frequency, and hence the corresponding frequency difference between them is passively stable around a few hundred hertz within millihertz fluctuation. While greatly reducing the size, complexity, and cost of the laser source by use of a single free-running fibre laser, the dual THz comb spectroscopy system maintains a spectral bandwidth and dynamic range of spectral power comparable to a system equipped with dual stabilized fibre lasers, and can be effectively applied to high-precision spectroscopy of acetonitrile gas at atmospheric pressure. The demonstrated results indicate that this system is an attractive solution for practical applications of THz spectroscopy and other applications.

## Introduction

Terahertz (THz) frequency combs have attracted attention as precise frequency scales of broadband THz radiation in THz frequency metrology^1,2^. A THz comb possesses the characteristics of both broadband radiation and narrow-linewidth radiation due to its comb-tooth-like spectrum, and an absolute frequency of its all modes is secured by a frequency standard via a coherent frequency-comb link. Such frequency traceability to the frequency standard is a significant advantage of THz comb, lacking in conventional THz frequency metrology. While high potential of THz combs has been used for the absolute frequency measurement of continuous-wave THz source^3–6^, it has been further extended to spectroscopy by dual THz comb spectroscopy (THz-DCS)^7–10^. In THz-DCS, based on multi-frequency heterodyning^7,11,12^ or asynchronous optical sampling (ASOPS)^13–15^ of dual THz combs with different frequency spacings (=*f*_*rep1*_, *f*_*rep2*_), the frequency scale of THz combs has been downscaled by a ratio of *f*_*rep2*_*/∆f*_*rep*_ to the radio-frequency (RF) region as an RF comb with a frequency spacing of *∆f*_*rep*_ (=*f*_*rep1*_ − *f*_*rep2*_). This enables us to obtain a mode-resolved THz comb spectrum (see Methods). Its utility has been demonstrated in high-precision broadband spectroscopy of polar molecular gas^16–18^, and its performance was enhanced to Doppler-limited spectroscopy with kHz linewidth^19^. However, the use of a pair of dual femtosecond lasers and the requirement for stabilization control of their repetition frequencies or frequency spacing (*f*_*rep1*_, *f*_*rep2*_) and/or its difference (*∆f*_*rep*_) hamper the practical the use of THz-DCS because of the difficulty of implementation and cost involved.

An interesting approach to simplify THz-DCS is the use of THz quantum cascade laser (QCL)^20,21^ because of high power and on-chip compactness. However, since a frequency spacing of THz-QCL comb is typically a few tens GHz, the reduced number of THz comb modes and too discrete mode distribution might limit its application for high-precision broadband spectroscopy. Another interesting approach is the use of electro-optics modulator (EOM)^22^. EOM-based THz-DCS has the advantage of stable and tunable *f*_*rep*_ and *∆f*_*rep*_; however, its spectral bandwidth is limited to less a few tens GHz by the electric frequency bandwidth of EOM. As for dual comb spectroscopy in infrared region, in addition to QCL^23^, EOM^24^, micro-resonator comb^25^, waveguide laser^26^, and semiconductor disk laser^27^, single-cavity dual-comb lasers have attracted attention as a low-cost, low-complexity, DCS sources because they did not need dual cavities and their stabilization control. Single-cavity dual-comb lasers are operated by multiplexing mode-locking operations in the wavelength^28,29^, polarization^30,31^, or propagation directions^32–34^. Its utility has been demonstrated in pump-probe measurement^35^, ranging^36^, and spectroscopy^28^ in the near-infrared region. If such single-cavity dual-comb lasers are implemented in THz-DCS, its practicability will be greatly enhanced. However, there are no attempts to implement single-cavity dual-comb lasers in THz-DCS. This is because THz-DCS needs dual THz combs with significantly small difference of frequency spacing (typically, several tens to a few hundred Hz)^7–10,16–19^ and hence the single-cavity dual-comb laser has to be optimized for THz-DCS.

**Figure 1:**
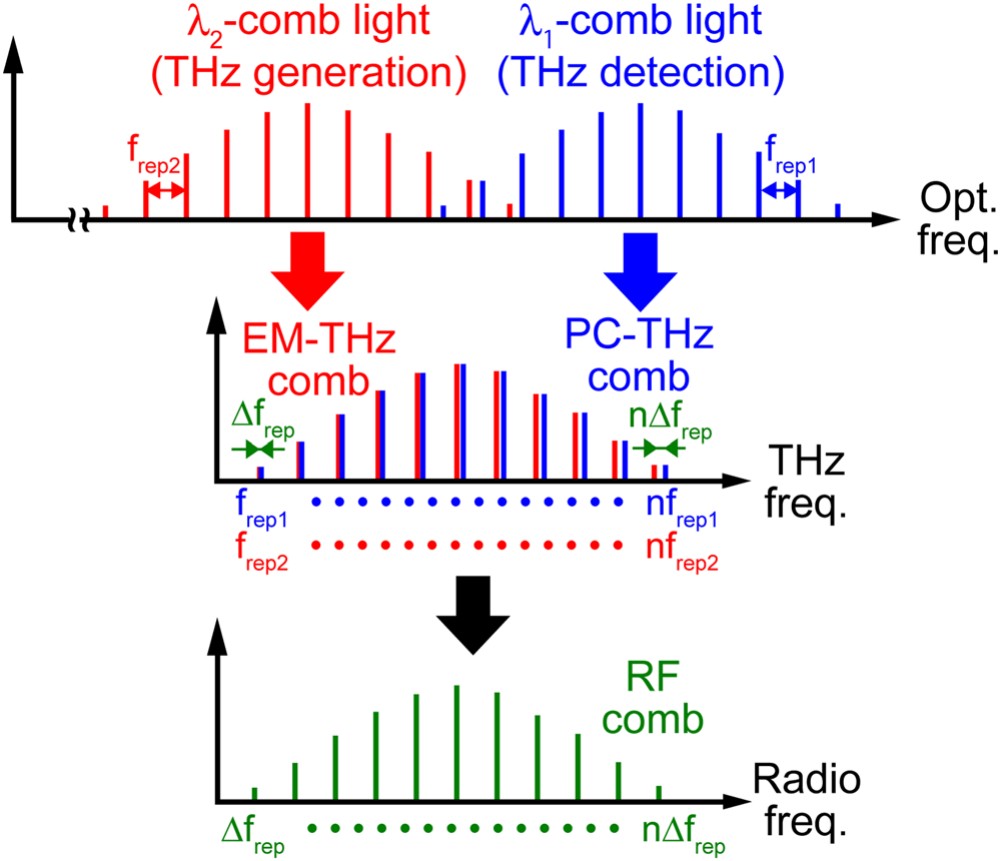
Principle of dual THz comb spectroscopy. λ_1_-comb light (frequency spacing = *f*_*rep1*_) is incident on a photoconductive antenna for THz detection (PCA detector), resulting in generation of a photocarrier THz comb (PC-THz comb, frequency spacing = *f*_*rep1*_) in the PCA detector. λ_2_-comb light (frequency spacing = *f*_*rep2*_) is incident on a photoconductive antenna for THz generation (PCA emitter), resulting in radiation of an electromagnetic THz comb (EM-THz comb, frequency spacing = *f*_*rep2*_) from the PCA emitter. When the free-space-propagating EM-THz comb is detected by the PCA detector having the PC-THz comb, a secondary frequency comb in the RF region (RF comb, frequency spacing = *∆f*_*rep*_ = *f*_*rep1*_ − *f*_*rep2*_) is generated as a current signal from the PCA detector via multi-frequency-heterodyning photoconductive detection between the EM-THz comb and PC-THz comb. The RF comb is a replica of the EM-THz comb whose frequency spacing is downscaled from *f*_*rep2*_ to *∆f*_*rep*_ by a conversion factor *f*_*rep2*_/*∆f*_*rep*_.

In this article, we demonstrate THz-DCS by simply replacing dual stabilized lasers with a single free-running dual-wavelength-comb (dual-λ-comb) Er-doped fibre (Er:fibre) laser^28,29^ without the need for additional apparatuses. Two independent mode-locked oscillations in different wavelength regions are multiplexed in a single fibre cavity (upper row in Fig. 1). The dual-λ-comb light beams have different repetition frequencies, *f*_*rep1*_ and *f*_*rep2*_, due to wavelength dispersion of the cavity fibre, and their frequency spacing fluctuates in a common-mode manner due to sharing of the same cavity. Such common-mode fluctuation leads to high stability in *∆f*_*rep*_ without the need for active laser stabilization. To optimize for THz-DCS, Δ*f*_*rep*_ value is significantly reduced down to a few hundred Hz, which is 10% of typical Δ*f*_*rep*_ in single-cavity dual-comb lasers, by precise adjustment of the cavity dispersion and length. We generate dual THz combs with different frequency spacings by a combination of dual-λ-comb light beams and photoconductive antennae (middle row in Fig. 1), and apply the resulting RF comb (lower row in Fig. 1) to spectroscopy of molecular gas.

## Results

**Figure 2:**
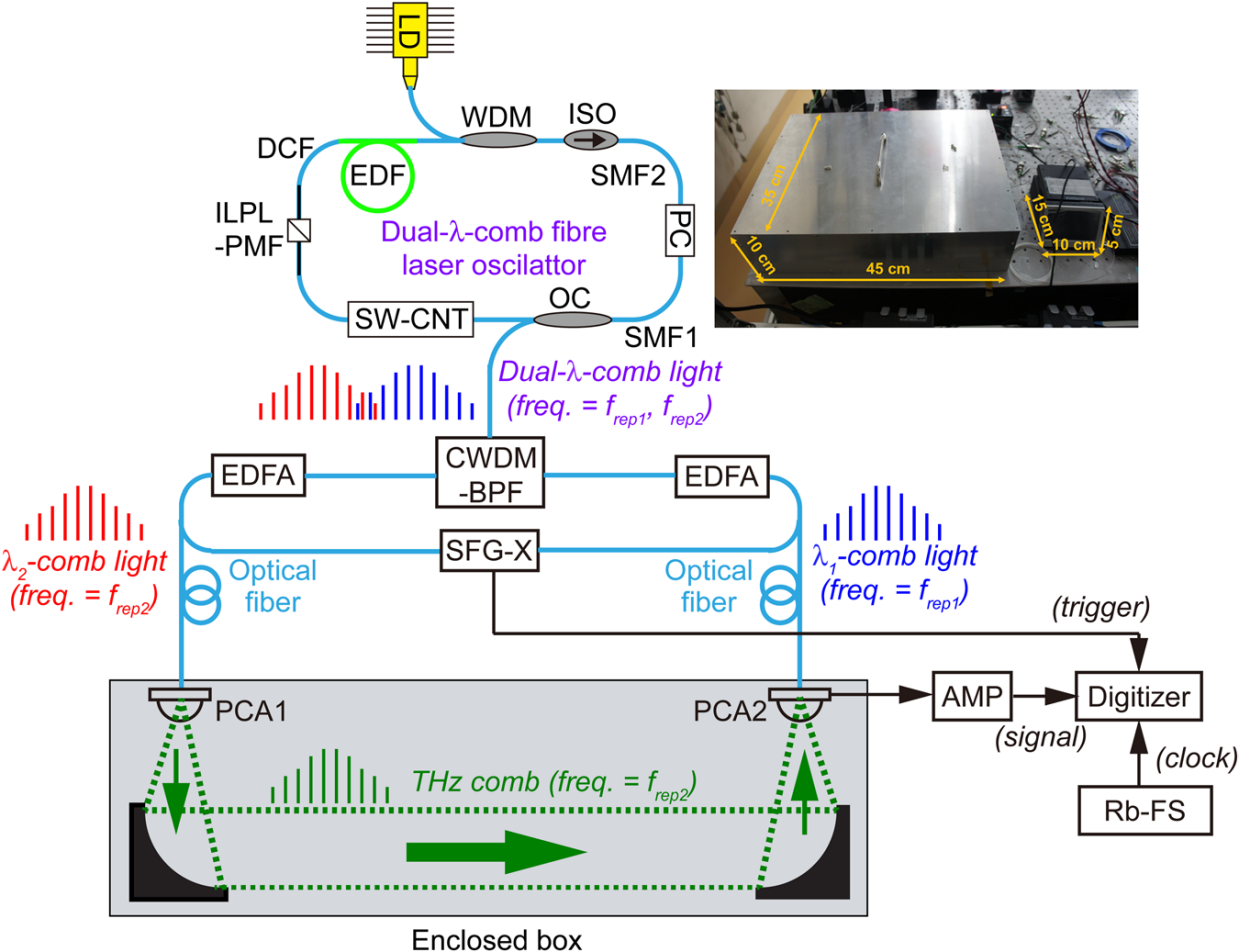
Experimental setup. An inset shows an optical photograph of dual-λ-comb fibre laser oscillator. See Methods for details.

Figure 2 illustrates the experimental setup of the dual-λ-comb fibre laser and the THz-DCS system, which describes in the Methods section together with details of the experimental and analytical methodology employed for the following measurements. We briefly describe three essential points that are important in the present experimental setup. First, an in-line polarizer with polarization-maintained fibre pigtails (ILPL-PMF) functions as an intra-cavity Lyot filter. Dual mode-locking oscillations were independently achieved at different wavelengths by separating two peaks of the gain profile in erbium-doped fibre with the Lyot filter. Second, a coarse-wavelength-division-multiplexing bandpass filter (CWDM-BPF) separates the spatially-overlapped dual-λ-comb lights into short-wavelength λ_1_-comb light (centre wavelength = 1533 nm) and long-wavelength λ_2_-comb light (centre wavelength = 1543 nm) for fibre amplification and THz-DCS. Third, a sum-frequency-generation cross-correlator (SFG-X) provides a time origin signal to acquire the temporal waveform in ASOSP measurement^14–19^, which is used as a trigger signal for the digitizer.

**Figure 3:**
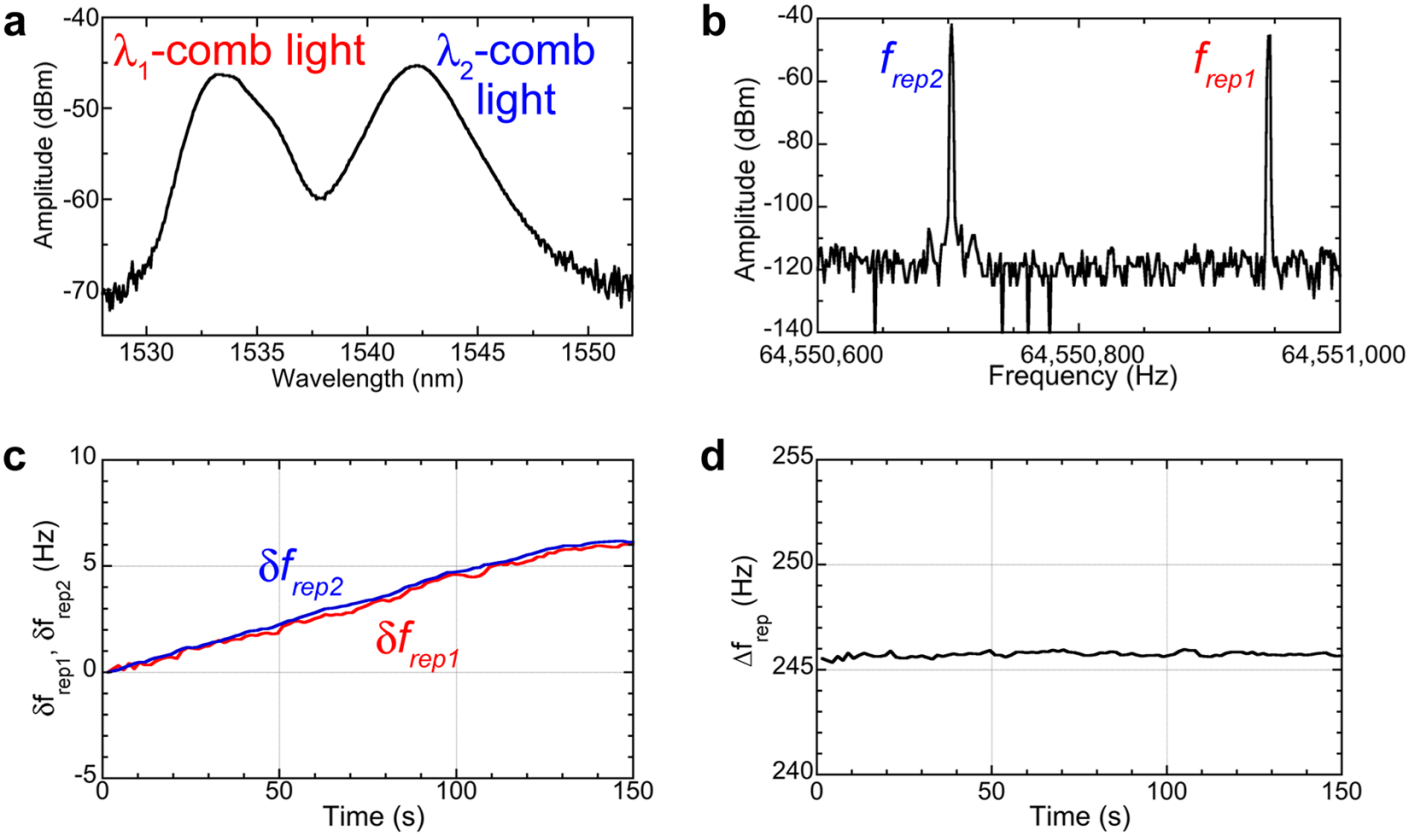
Basic performance of dual-λ-comb Er:fibre laser oscillator light. (**a**) Optical spectrum of dual-λ-comb light. (**b**) RF spectra of repetition frequency signal in dual-λ-comb light. (**c**) Temporal fluctuations of *δf*_*rep1*_ and *δf*_*rep2*_. (**d**) Temporal fluctuations of *∆f*_*rep*_.

First, we evaluated the basic performance of dual-λ-comb light radiating from the fibre laser oscillator. Figure 3a shows the optical spectra of the dual-λ-comb light: λ_1_-comb light with a centre wavelength of 1533 nm and λ_2_-comb light with a centre wavelength of 1543 nm. Although the spectral tails of the λ_1_-comb light and λ_2_-comb light were somewhat overlapped around 1537.5 nm, these comb light beams were independently mode-locked without coupling to each other. Evidence for two independent mode-locking oscillations was confirmed in the RF spectrum of the dual-λ-comb light (Fig. 3b): two frequency spikes appeared around 64.55 MHz, corresponding to different repetition frequencies of the dual-λ-comb light. Due to the low anomalous dispersion of the cavity fibre around this wavelength region, the λ_1_-comb light had a higher repetition frequency *f*_*rep1*_, whereas the λ_2_-comb light had a lower repetition frequency *f*_*rep2*_, and the frequency difference *∆f*_*rep*_ between them was considerably small (~ a few hundred Hz). We also evaluated the temporal fluctuations of *f*_*rep1*_, *f*_*rep2*_, and *∆f*_*rep*_ because they determine the frequency-scale conversion factor (=*f*_*rep2*_/*∆f*_*rep*_) between the THz comb and the RF comb (see Fig. 1 and Methods)^7^. Figure 3c shows the temporal behaviour of the deviation in *f*_*rep1*_ and *f*_*rep2*_ (*δf*_*rep1*_ and *δf*_*rep2*_) from initial values with respect to the elapsed time. Due to the free-running or unstabilized operation, *f*_*rep1*_ and *f*_*rep2*_ showed a slow drift behaviour of several Hz within a range of 150 s. More importantly, these fluctuations were in common due to the common-path cavity. This resulted in millihertz fluctuation of *∆f*_*rep*_ without drift behaviour (Fig. 3d) even though there are no active laser stabilization.

**Figure 4:**
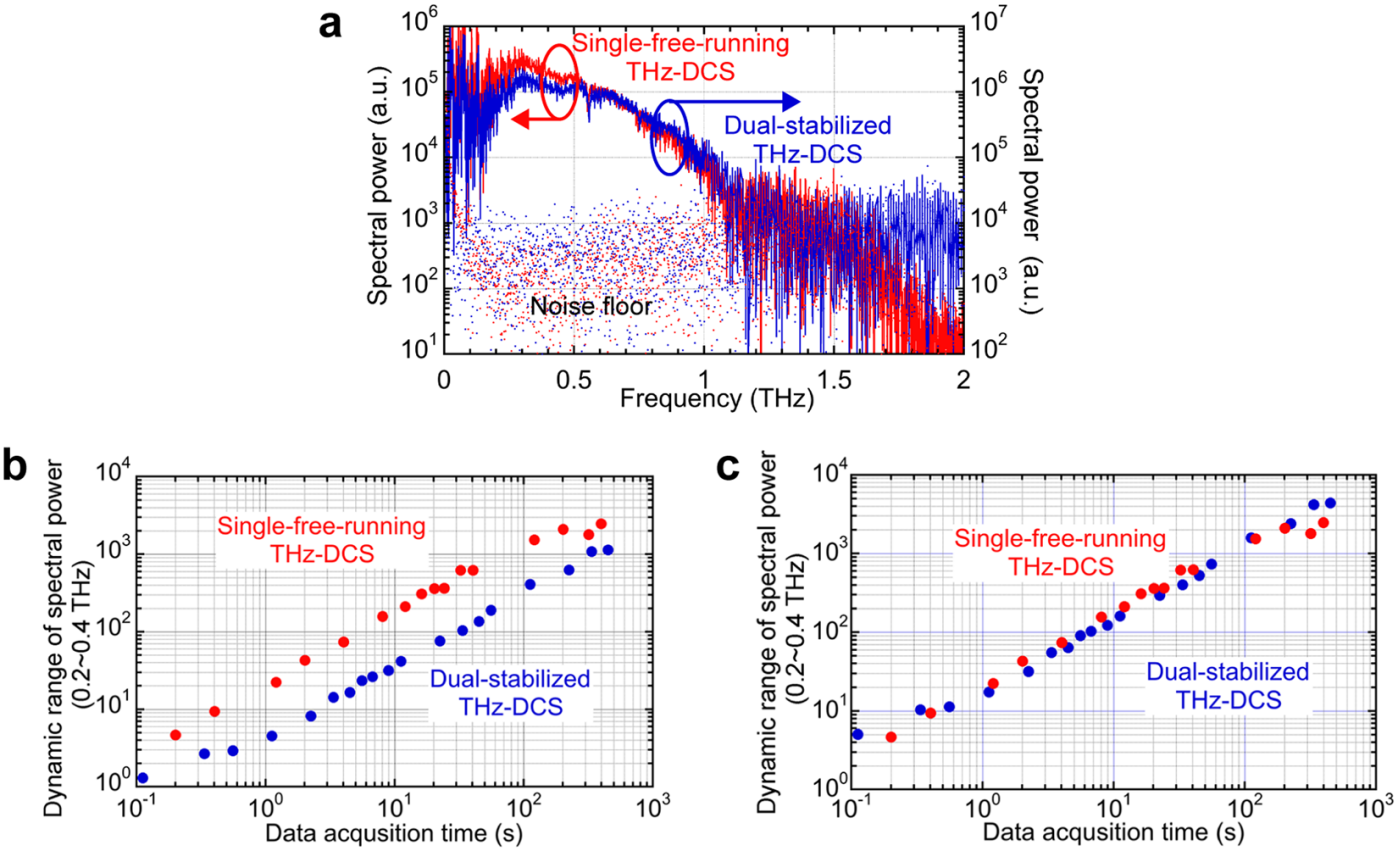
Comparison of basic performance between single-free-running and dual-stabilized THz-DCS systems. (**a**) Spectral bandwidth. Dynamic range of THz spectral power (**b**) before and (**c**) after correction of different repetition frequencies.

We next evaluated the basic performance of the present THz-DCS system equipped with a single free-running dual-λ-comb Er:fibre laser (*f*_*rep1*_ ≈ *f*_*rep2*_ ≈ 64.55 MHz, *∆f*_*rep*_ = 248.4 Hz), namely, a single-free-running THz-DCS system, by comparing it with our previous THz-DCS system equipped with dual stabilized Er:fibre lasers (*f*_*rep1*_ ≈ *f*_*rep2*_ ≈ 250.00 MHz, *∆f*_*rep*_ = 893.00 Hz, see Methods)^9,10^, namely, a dual-stabilized THz-DCS system. Both THz-DCS systems used the same THz optical systems and data acquisition electronics. Figure 4a shows a comparison of the THz power spectrum between the single-free-running and dual-stabilized THz-DCS systems when the data acquisition time was set to 100 s. The spectral resolution was set to 1 GHz. For reference, the noise spectrum is shown in the same graph. The spectral bandwidth and dynamic range of spectral power were comparable to each other. We also investigated the relation between the data acquisition time and dynamic range of spectral power within a frequency range of 0.2 to 0.4 THz (see Fig. 4b). The linear relationship between them clearly indicated that the timing jitter in both systems had little influence. Also, the dynamic range over 10 was achieved at the data acquisition time of 1 s. Although the dynamic range in the single-free-running THz-DCS system was higher than that in the dual-stabilized THz-DCS systems, this difference was mainly due to difference of repetition frequencies between them. If such difference is corrected, both were almost overlapped (see Fig. 4c). In this way, the passive stabilization of *∆f*_*rep*_ in the free-running dual-λ-comb Er:fibre laser was as powerful as the active stabilization of *f*_rep1_ and *f*_*rep2*_ in the dual stabilized Er:fibre lasers.

**Figure 5:**
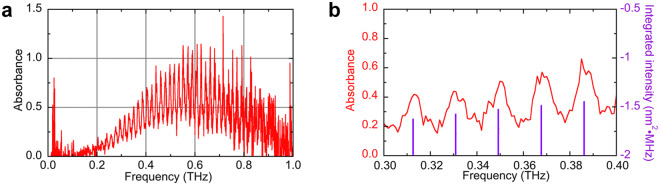
Spectroscopy of CH_3_CN gas in the atmospheric pressure. (**a**) Absorbance spectrum of CH_3_CN gas within a frequency range of 0 to 1 THz. (**b**) Magnified absorbance spectrum of CH_3_CN gas within a frequency range of 0.3 to 0.4 THz. Purple lines show literature values of integrated intensity for CH_3_CN gas in the JPL database^41^.

Finally, we demonstrated THz spectroscopy of acetonitrile (CH_3_CN) gas under atmospheric pressure. Since CH_3_CN gas is contained in the interstellar medium^37^, incomplete combustion gas of nylon textiles^38^, volatile organic compounds related with atmospheric pollution and biomarkers^39^, it is important to perform THz spectroscopy of this molecular gas in astronomy, fire accidents, atmospheric analysis, and health monitoring. Due to the symmetric top molecule, CH_3_CN gas exhibits characteristic spectral fingerprints with GHz structure in the THz region under atmospheric pressure: a series of manifolds of multiple rotational transitions regularly spaced by *2B*, where *B* is the rotational constant (=9.194 GHz)^40^. After an enclosed box for THz optics was filled with CH_3_CN gas at atmospheric pressure, the THz power spectrum was acquired by the single-free-running THz-DCS system. Figure 5a shows the absorbance spectrum of CH_3_CN gas (*f*_*rep1*_ ≈ *f*_*rep2*_ ≈ 64.55 MHz, *∆f*_*rep*_ = 388.6 Hz, spectral resolution =1 GHz, data acquisition time = 257 s). Thirty-nine manifolds of absorption lines periodically appeared with a constant frequency separation, and could be assigned to rotational quantum numbers from *J* = 15 around 0.29 THz to *J* = 53 around 0.98 THz correctly. Figure 5b shows the magnified absorbance spectrum of the same CH_3_CN gas within a frequency range of 0.3 to 0.4 THz. For reference, the literature values of integrated intensity for CH_3_CN gas in the JPL spectral database^41^ is shown as purple lines in Fig. 5b. Although neighbouring manifolds of rotational transitions were somewhat overlapped due to the pressure broadening at atmospheric pressure, the spectra show similar signatures to the literature values, namely, a periodic structure of absorption peaks with a constant spacing exactly equal to *2B* (=18.4 GHz). In this way, we confirmed the effectiveness of the single-free-running THz-DCS for atmospheric-pressure gas spectroscopy in the THz region.

## Discussion

**Figure 6:**
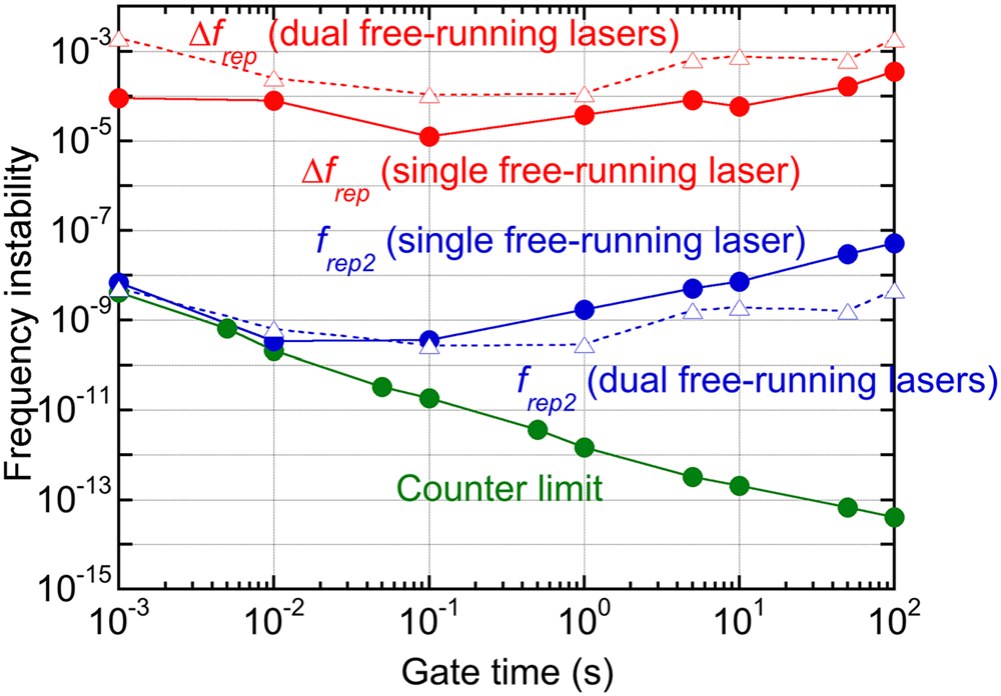
Comparison of frequency instability in *f*_*rep2*_ and ∆*f*_*rep*_ with respect to gate time for single free-running dual-λ-comb Er:fibre laser and dual stabilized Er:fibre lasers. Limit of an RF frequency counter (53220 A, Keysight Technologies) is indicated as green circle plots.

We confirmed that the use of the single free-running fibre laser did not degrade the spectroscopic performance of THz-DCS, and that the single-free-running THz-DCS system has the potential for atmospheric-pressure gas spectroscopy. Here, we first discuss the limitations of spectral resolution and accuracy. The spectra resolution was adjusted to 1 GHz in this article to fit the pressure broadening linewidth of several GHz by setting the time window size of 1 ns in the temporal waveform of the pulsed THz radiation. It is important to note that the present THz-DCS system still has space to decrease the spectral resolution down to *f*_*rep2*_ (≈64.55 MHz) by full use of the whole time window size (=1/*f*_*rep2*_). Also, combined use of the spectral interleaving method^10^ or frequency division of *f*_*rep1*_ and *f*_*rep2*_^42^ will reduce the spectral sampling interval down to MHz order. On the other hand, the instability of the conversion factor limits the spectral accuracy because the frequency scale of the THz comb was decreased to the RF region based on a conversion factor of *f*_*rep2*_/*∆f*_*rep*_. Blue and red circle plots in Fig. 6 show the frequency instability of *f*_*rep2*_ and *∆f*_*rep*_ for the single free-running fibre laser, respectively. The frequency instability was defined as the ratio of Allan deviation to the mean of *f*_*rep2*_ or *∆f*_*rep*_. The instability of the conversion factor is essentially limited by that of *∆f*_*rep*_ rather than *f*_*rep2*_, and was achieved 10^−4^ in the data acquisition time of one hundred seconds. This value directly corresponds to the spectral accuracy in THz-DCS. The spectral accuracy of 10^−4^ is sufficient for most of THz spectroscopic applications including the atmospheric-pressure gas spectroscopy^18^ (pressure-limited absorption linewidth > a few GHz); however, it may be insufficient for high-precision spectroscopy, such as the low-pressure gas spectroscopy^10^ (Doppler-limited absorption linewidth > a few MHz). To enhance the spectral accuracy in the single-free-running THz-DCS up to the requirement for high-precision spectroscopy, the adaptive sampling method^43^ is a promising approach based on powerful correction of the laser timing jitter. Work is in progress to combine the single-free-running THz-DCS and the adaptive sampling method.

It is interesting to compare the frequency instability of *f*_*rep2*_ and *∆f*_*rep*_ between the single free-running laser and dual free-running lasers. To this end, we evaluated the frequency instability of them when dual stabilized Er:fibre lasers (see Methods) were operated without the frequency stabilziation, namely dual free-running lasers. Blue and red triangle plots in Fig. 6 respectively show the frequency instability of *f*_*rep2*_ and *∆f*_*rep*_ for the dual Er:fibre lasers without frequency stabilization. The *f*_*rep2*_ instability of the single free-running laser (blue circle plots in Fig. 6) was a little worse than that of the dual free-running lasers (blue triangle plots in Fig. 6) at longer gate time because the temperature control of the fibre cavity was not adopted for the single free-running laser. However, the *∆f*_*rep*_ instability of the single free-running laser (red circle plots in Fig. 6) was significantly better than that of the dual free-running lasers (red triangle plots in Fig. 6). This comparison indicates the advantage to sharing of the same cavity even under no temperature control of the cavity.

Next, we discuss the practicability of the single-free-running THz-DCS system. Compared with the dual-stabilized THz-DCS system, use of the single free-running fibre laser largely enhances the practicability from the viewpoints of compactness, cost effectiveness, and ease of implementation of the laser source. An inset of Fig. 2 shows the optical photograph of the single free-running dual-λ-comb Er:fibre laser, including a single laser oscillator (size: 450 mm width × 350 mm depth × 100 mm height), EDFAs (size: 150 mm width × 120 mm depth × 20 mm height) and a LD driver (size: 100 mm width × 150 mm depth × 50 mm height). On the other hand, dual stabilized Er:fibre lasers are composed of dual laser heads (size: 415 mm width × 400 mm depth × 110 mm height) and control electronics (mounted in a 19″ rack cabinet, size: 600 mm width × 800 mm depth × 1800 mm height). Although the size of the dual-λ-comb Er:fibre laser head is comparable to that of dual stabilized Er:fibre laser heads, there are still space to largely reduce its size in the former due to the simple cavity configuration without the need for laser control (see Fig. 2). Regarding the accompanying electronics, the total volume of the dual-λ-comb Er:fibre laser was reduced to 0.086% of the volume of the dual stabilized Er:fibre lasers. Such a large reduction of the laser size will lead to the development of portable THz-DCS systems with flexible and robust fibre-coupled photoconductive antennae. Also, the lack of need for laser stabilization electronics drastically decreases the cost and complexity of the laser source as well as its size. In this way, despite the fact that the single-free-running THz-DCS system uses a much more compact, more cost-effective, and more easy-to-operate laser than the dual-stabilized THz-DCS system, its spectroscopic performance is comparable to that of the previous THz-DCS system.

In conclusion, we greatly enhanced the versatility of THz-DCS by use of a single free-running dual-λ-comb Er:fibre laser without degrading its high performance in terms of spectral resolution, spectral accuracy, broad spectral bandwidth, and data acquisition time. By using a cavity configuration similar to that of usual free-running mode-locked fibre lasers, dual-λ-comb light beams with passively stable *∆f*_*rep*_ were generated via wavelength-region multiplexing of mode-locked oscillations in the same cavity. Use of such dual-λ-comb light beams in THz-DCS will enable a drastic reduction in size, complexity, and cost. Although versatility always conflicts with high performance, this dual-λ-comb Er:fibre laser enables us to balance these competing characteristics in broadband high-precision THz spectroscopy, which is the first time to the best of our knowledge. The proposed versatile and high-performance THz-DCS will be a powerful tool as a simple, portable, universal, rapid THz spectrometer for gas analysis^18^, biosensor^44^, impulse radar^45^, and so on.

## Methods

### THz-DCS

A THz comb spectrum can be obtained by the multi-frequency-heterodyning method in the frequency domain (Fig. 1)^7,11,12^ or the ASOPS method in the time domain^13–15^. We here used the latter method. Supplementary Fig. 1 shows a signal flowchart of THz-DCS used here. The ASOPS method enabled us to linearly magnify the time scale of a THz pulse train having a repetition period of *1/f*_*rep2*_ based on a temporal magnification factor *f*_*rep2*_*/∆f*_*rep*_ to give an RF pulse train with a repetition period of *1/*∆f_rep_. The RF pulse train could be directly acquired by a digitizer without the need for mechanical time-delay scanning. The Fourier transform of this RF pulse train gave an RF comb spectrum with a frequency spacing of *∆f*_*rep*_. Finally, frequency calibration of the RF comb with the temporal calibration factor enabled us to obtain a THz comb spectrum with a frequency spacing of *f*_*rep2*_.

### Dual-λ-comb Er:fibre laser

The dual-λ-comb fibre laser oscillator had a ring cavity including a single-mode fibre (SMF1; SMF28, Corning, dispersion = 15.7 ps/nm/km at 1535 nm, length = 1.79 m), another single-mode fibre (SMF2; HI1060, Corning, dispersion = 5.6 ps/nm/km at 1535 nm, length = 0.11 m), a erbium-doped fibre (EDF; ER110-4/125, LIEKKI, dispersion = −9.6 ps/nm/km at 1535 nm, length = 0.56 m), a dispersion compensation fibre (DCF; DCF 180, Yangtze Optical Fibre and Cable Co., Ltd., dispersion = −180 ps/nm/km at 1535 nm, length = 0.145 m), a single-wall carbon nanotube mode-locker (SW-CNT), an in-line polarizer with polarization-maintained fibre pigtails (ILPL-PMF, dispersion of PMF = 15.7 ps/nm/km at 1535 nm, length of PMF = 0.6 m), a polarization controller (PC), an wavelength division multiplex (WDM), an isolator (ISO), an output coupler (OC), and a pump laser diode (LD, wavelength = 980 nm), as shown in Fig. 2. The key point in dual-λ-comb fibre laser oscillator is that two independent mode-locked oscillations were achieved at different wavelengths in a single fibre cavity. To this end, we first adjusted the gain profile of the EDF to show double spectral peaks centred around 1533 nm and 1543 nm by intracavity loss tuning with the pump power and PC. Such close spectral peaks enable much smaller *∆f*_*rep*_ than that in previous dual-λ-comb fibre lasers^28,29^. To separate the double-peak spectrum as two independent spectra, the birefringence-induced spectral filtering (Lyot filtering) was adopted in the cavity by the ILPL-PMF. Under this condition, SW-CNT activated dual-λ mode-locking oscillation. Our previous dual-λ-comb Er:fibre lasers provided a stable Δ*f*_*rep*_ around a few kHz; however, this Δ*f*_*rep*_ value is too high for THz-DCS because the high Δ*f*_*rep*_ value leads to a small temporal magnification factor (=*f*_*rep2*_*/*∆*f*_*rep*_). We reduced the total dispersion to 7 fs/nm/km in a cavity length of 3.2 m by a combination of SMF, EDF, and DCF, and achieved the Δ*f*_*rep*_ value much lower than that of the previous laser. Output light beams from the dual-λ-comb fibre laser oscillator were separated into short-wavelength λ_1_-comb light (centre wavelength = 1533 nm) and long-wavelength λ_2_-comb light (centre wavelength = 1543 nm) by a coarse-wavelength-division-multiplexing bandpass filter (CWDM-BPF, passband = 1530 ± 7.5 nm). Supplementary Fig. 2a shows optical spectra of the spectrally separated λ_1_-comb light and λ_2_-comb light. The resulting λ_1_-comb light and λ_2_-comb light were amplified and spectrally broadened by a pair of Er:fibre amplifiers (EDFAs). Then, their pulse duration was minimized by dispersion control with SMF and DCF. The λ_1_-comb light (centre wavelength = 1531 nm, mean power = 20 mW, and pulse duration = 130 fs) was used for probe light, whereas the λ_2_-comb light (centre wavelength = 1543 nm, mean power = 27 mW, and pulse duration = 130 fs) was used for pump light in the THz-DCS system. Supplementary Fig. 2b shows optical spectra of the amplified λ_1_-comb light and λ_2_-comb light. These spectra were not completely overlapped with each other; however, some difference of the centre wavelength did not influence THz-DCS if the spectral bandwidth is similar to each other. This is because such difference is negligible in generation and detection of THz comb with photoconductive antenna (PCA). Only photon energy that exceed a bandgap energy of PCA material is required for the amplified λ_1_-comb light and λ_2_-comb light; the 1.5-µm light satisfies this requirement in the present PCA. Supplementary Fig. 2c,d show their auto-correlation trace. Such pulse duration is sufficient for THz-DCS.

### Dual stabilized Er:fibre lasers

Dual *f*_*rep*_-stabilized, mode-locked Er-fibre lasers (ASOPS TWIN 250, Menlo Systems, centre wavelength = 1550 nm, pulse duration = 50 fs, *f*_*rep1*_ ≈ *f*_*rep2*_ ≈ 250 MHz) were used in the dual-stabilized THz-DCS system for comparison with the single-free-running THz-DCS system. The frequencies *f*_*rep1*_ and *f*_*rep2*_ were stabilized at 250,000,893 Hz and 250,000,000 Hz by two independent laser control systems (RMS timing jitter < 150 fs in the range 0.1 Hz–500 kHz) referenced to a rubidium frequency standard (Rb-FS; FS725, Stanford Research Systems, accuracy = 5 × 10^−11^ and instability = 2 × 10^−11^ at 1 s). Thus, the frequency difference between them (*∆f*_*rep*_ = *f*_*rep1*_ − *f*_*rep2*_ = 893 Hz) was indirectly stabilized, too. These values of *f*_*rep2*_ and *∆f*_*rep*_ give the same TMF as the single-free-running THz-DCS system, enabling us to use the same data acquisition electronics in both systems. The *f*_*rep2*_ light was used for pump light, whereas the *f*_*rep1*_ light was used for probe light in THz-DCS system.

### Experimental setup

A pair of fibre-coupled LT-InGaAs/InAlAs PCAs was used for the THz-DCS system: a strip-line-shaped LT-InGaAs/InAlAs PCA (PCA1, TERA 15-TX-FC, Menlo Systems, bias voltage = 20 V, optical power = 20 mW) for a THz emitter and a dipole-shaped LT-InGaAs/InAlAs PCA (PCA2, TERA 15-RX-FC, Menlo Systems, optical power = 20 mW) for a THz detector. THz pulse train was radiated from the PCA1 triggered by the λ_2_-comb light (pump light), propagated in an enclosed box for THz optics, and was then incident on PCA2 together with the λ_1_-comb light (probe light) for ASOPS measurement (see an upper part in Supplementary Fig. 1). The enclosed box was filled with nitrogen gas or acetonitrile gas. Since a temporal waveform of the output current signal from PCA2 was corresponding to that of THz electric field, the temporal waveform of PCA2 output current was acquired as the RF pulse train by a digitizer (National Instruments, PCI-5122, sampling rate = 1 × 10^8^ samples/s, resolution = 14 bit) after amplification with a current preamplifier (AMP; FEMTO Messtechnik GmbH, HCA-10M-100K, bandwidth = 10 MHz, trans-impedance gain = 1 × 10^5^ V/A). Portions of the λ_1_-comb light and the λ_2_-comb light separated by OCs were fed into a sum-frequency-generation cross-correlator (SFG-X) for generation of a trigger signal for the digitizer. A rubidium frequency standard (Rb-FS; FS725, Stanford Research Systems, accuracy = 5 × 10^−11^ and instability = 2 × 10^−11^ at 1 s) was used for an external clock signal in the digitizer. The sampling interval and time window size were respectively set to 60 fs and 15.5 ns for the single-free-running THz-DCS system and to 14 fs and 4 ns for the dual-stabilized THz-DCS system.

### Data analysis

We set the spectral resolution of the THz-DCS system to be 1 GHz to fit it with the pressure-broadening absorption linewidth. To this end, a temporal waveform of the pulsed THz radiation was extracted with a time window size of 1 ns from the whole temporal waveform of the pulsed THz radiation. Then, the THz power spectrum was obtained by taking the Fourier transform of the temporal waveform and subsequent squaring, and the result was used for calculation of the absorbance spectrum. The absorbance spectrum was obtained by using the THz power spectrum obtained when the enclosed box was filled with nitrogen gas as a reference.

### Data availability

The data that support the findings of this study are available from the corresponding author upon reasonable request.

## Electronic supplementary material

Supplementary Information
