# Supplementary material for: Dual terahertz comb spectroscopy with a single free-running fibre laser
Source: Sci Rep. 2018 Jul 24;8:11155. doi: 10.1038/s41598-018-29403-9 (PMC6057945; doi:10.1038/s41598-018-29403-9)
Supplement: Supplementary file 1 — Supplementary Information [file 41598_2018_29403_MOESM1_ESM.docx]

**Dual terahertz comb spectroscopy with a single free-running fibre laser**

Guoqing Hu^1,2^, Tatsuya Mizuguchi^1,3^, Ryo Oe^1,3^, Kazuki Nitta^1^, Xin Zhao^2^, Takeo Minamikawa^3,4^, Ting Li^2^, Zheng Zheng^2,5^, and Takeshi Yasui^3,4^

*^1^Graduate School of Advanced Technology and Science, Tokushima University, 2-1, Minami-Josanjima, Tokushima, Tokushima 770-8506, Japan*

*^2^School of Electronic and Information Engineering, Beihang University, Beijing, 100191, China*

*^3^JST, ERATO, MINOSHIMA Intelligent Optical Synthesizer Project, 2-1, Minami-Josanjima, Tokushima, Tokushima 770-8506, Japan*

*^4^Graduate School of Technology, Industrial and Social Sciences, Tokushima University, 2-1, Minami-Josanjima, Tokushima, Tokushima 770-8506, Japan*

*^5^Collaborative Innovation Centre of Geospatial Technology, 129 Luoyu Road, Wuhan 430079, China*

*^*^Correspondence to: yasui.takeshi@tokushima-u.ac.jp*

This file includes:

Supplementary Figure 1

Supplementary Figure 2


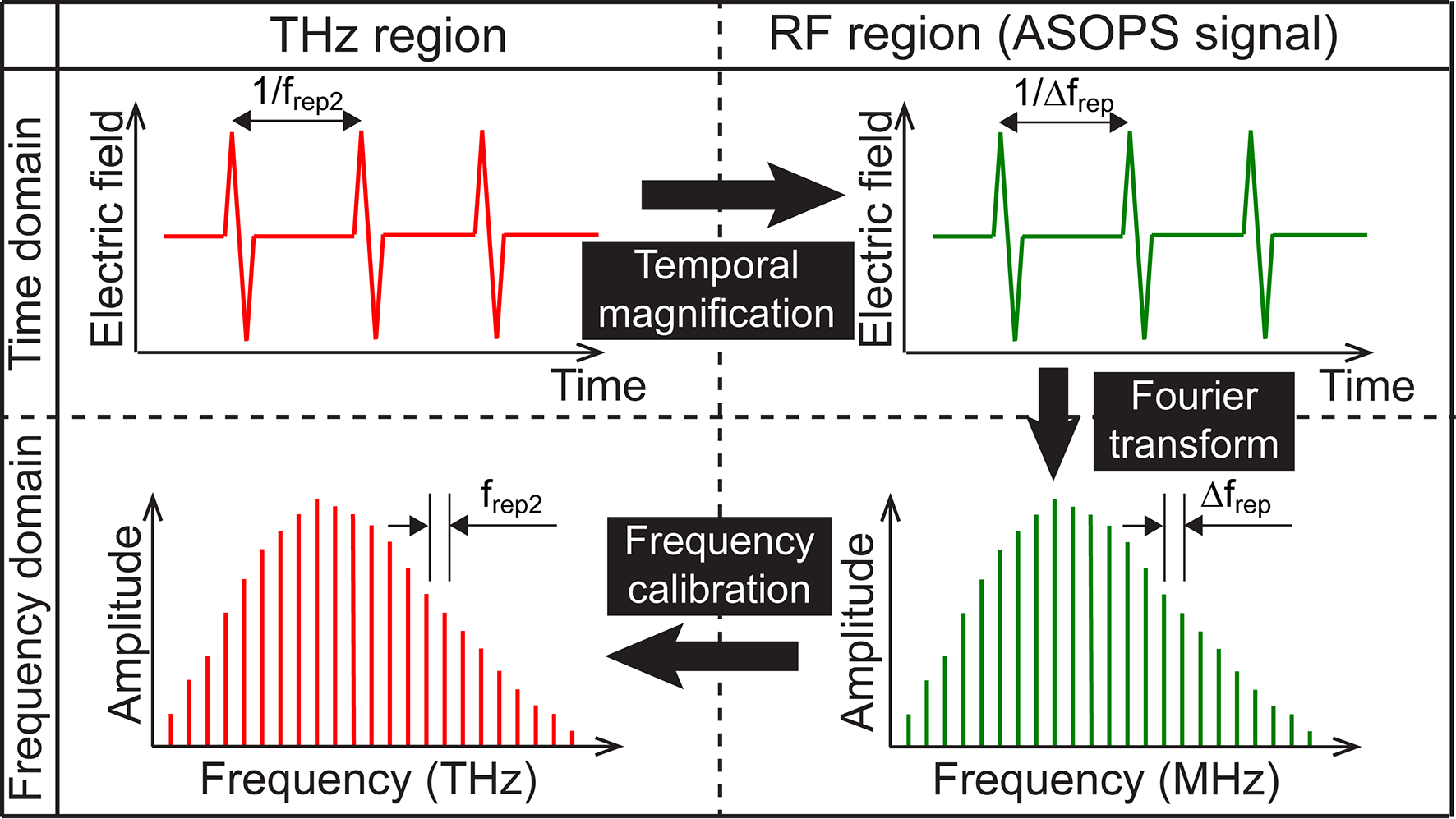


Supplementary Fig. 1. **Principle of THz-DCS in time domain.** See Methods for details.


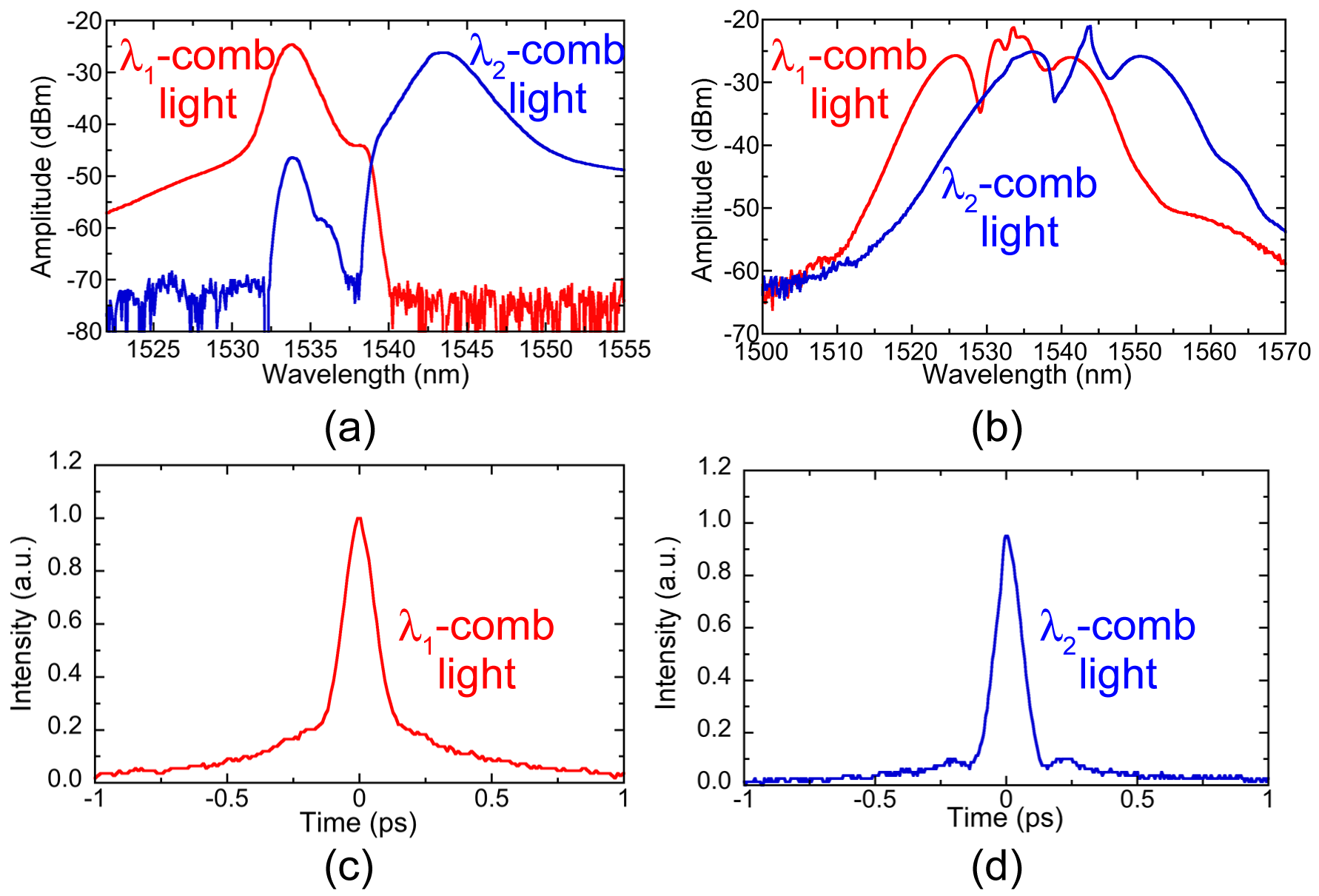


Supplementary Fig. 2. **Basic performance of spectrally separated and amplified dual-**λ**-comb Er:fibre laser lights.** **a**, Optical spectra of separated λ_1_-comb light and λ_2_-comb light. **b**, Optical spectra of amplified λ_1_-comb light and λ_2_-comb light. **c**, Auto-correlation trace of amplified λ_1_-comb light. **d**, Auto-correlation trace of amplified λ_2_-comb light.
